# Innate generation of thrombin and intracellular oxidants in airway epithelium by allergen Der p 1

**DOI:** 10.1016/j.jaci.2016.05.006

**Published:** 2016-10

**Authors:** Jihui Zhang, Jie Chen, Kimberley Allen-Philbey, Chathuri Perera Baruhupolage, Theresa Tachie-Menson, Shannon C. Mangat, David R. Garrod, Clive Robinson

**Affiliations:** a Institute for Infection & Immunity, St George's, University of London, London, United Kingdom; b Faculty of Life Sciences, University of Manchester, Manchester, United Kingdom

To the Editor:

Group 1 cysteine protease allergens from house dust mites (HDMs) are targets of a new class of drugs known as allergen delivery inhibitors (ADIs), which are entering development for asthma therapy.^1^ In studying pro-inflammatory signaling by protease allergens, attention has focused on their direct cleavage of protease-activated receptors (PARs).^2^ We have explored an alternative view, namely, that a key HDM allergen triggers the activation of thrombin and stimulates the production of intracellular reactive oxygen species (ROS) and the extracellular release of ATP. ROS have significance because they orchestrate an allergic polarization of immune responses, and both heightened ROS production and a broad deficit in antioxidant defenses are characteristics of asthma.^3^

To investigate the production of intracellular ROS we loaded human airway epithelial cells (primary cultures and established lines) with dihydrorhodamine 123 and exposed them to a natural mixture of *Dermatophagoides pteronyssinus* allergens. This resulted in a sustained generation of ROS (Fig 1, *A* and *B*) associated with mitochondria and nuclei (Fig 1, *C* and *D*; see the Methods section and Fig E1, *A*-*D*, in this article's Online Repository at www.jacionline.org).

ADZ 51,457 and ADZ 51,529, which are reversible ADIs targeting group 1 HDM protease allergens,^1^ substantially reduced ROS generation (Fig 1, *E*). Purified natural Der p 1 replicated ROS production and was fully inhibited by ADZ 51,457 (Fig 1, *F*). In contrast, an irreversible inhibitor of serine proteases had no effect on ROS production and purified Der p 2 conspicuously failed to elicit ROS generation (see Fig E2, *A* and *B*, in this article's Online Repository at www.jacionline.org). Thus, among natural HDM allergens, the initiators of intracellular ROS generation are the group 1 cysteine proteases.

Surprisingly, ROS production by HDM allergens was transduced through PAR1 and PAR4, with only a small contribution from PAR2 (Fig 1, *G* and *H*; see Fig E3, *A*-*E*, in this article's Online Repository at www.jacionline.org). These responses required the opening of pannexons, which are *inter alia* conduits for ATP release (Fig 1, *I*). Interestingly, the viral RNA surrogate polyinosinic:polycytidylic acid (poly i:c) also caused pannexon-dependent ROS production (Fig 1, *I*). Although HDM allergens and poly i:c initiated ROS production differently (see Fig E4, *A* and *B*, in this article's Online Repository at www.jacionline.org), their signaling converges at pannexons (Fig 1, *I*), with the extracellular release of ATP and activation of mechanisms sensitive to the allosteric P_2_X_7_ receptor modulator, AZ 10606120 (Fig 2, *A* and *B*).

Stimulation of PAR1 and PAR4 has not previously been associated with Der p 1,^2^ so we were interested in determining whether this involved the generation of thrombin, their canonical activator. The thrombin inhibitor argatroban inhibited ROS generation by HDM allergens, whereas the Factor Xa inhibitor apixaban was without effect (Fig 2, *C* and *D*; see Fig E5, *A*-*C*, in this article's Online Repository at www.jacionline.org), thus excluding thrombin formation by the full coagulation cascade. Interestingly, both argatroban and a PAR1 antagonist were effective inhibitors of poly i:c (Fig 2, *E* and *F*).

Incubation of prothrombin with mixed HDM allergens caused the appearance of prethrombin-1, the zymogen form of meizothrombin desF1, and the B chain of thrombin as major products. This process was inhibited by ADZ 50,000, an irreversible active site titrant analogue of ADZ 51,457 and ADZ 51,529^1^ (Fig 2, *G*). Formation of thrombin by Der p 1 provides further insight into the PAR siRNA data (Fig 1, *G* and *H*) and a possible explanation of the extensive antagonism of ROS formation by PAR1 antagonists (SCH 79797, FR 171113) and the PAR4 antagonist, tcY-NH_2_ (see Fig E3, *A*-*C*). Heterodimerization of PAR1 and PAR4 is precedented, providing a mechanism for thrombin bound to PAR1 through exosite 1 to cleave PAR4 (which cannot bind) more efficiently.^4^ The formation of a ternary complex would thus render ROS generation sensitive to antagonism of both receptors and imply that the main effector of Der p 1–stimulated ROS production might be PAR4, which is notably associated with epithelial-mesenchymal transition in airway cells.

Hitherto, PAR1 and PAR4 have not been considered activatable by group 1 HDM allergens,^2^ but in revealing the Der p 1–dependent cleavage of prothrombin we have identified their canonical activation with subsequent intracellular ROS formation via ATP release. Extracellular ATP is elevated in asthma, which is noteworthy because it stimulates dendritic cells and triggers the release of IL-33, which is genetically linked to asthma susceptibility and a key activator of cytokine production by iH_2_ nuocytes.^5^ Thrombin is present in airway surface liquid in asthma at levels sufficiently elevated to drive cell proliferation and is also increased following respiratory virus infection.^6^ Although it is generally assumed that these changes are associated with tissue repair following inflammation, our data implicate thrombin-mediated signaling as both an innate strategic initiator and an effector-perpetuator of allergic sensitization through its direct generation by inhaled Der p 1.

That the Toll-like receptor 3 ligand poly i:c operates ROS generation through a mechanism that converges with Der p 1 signaling at pannexons is interesting because interactions between allergens and respiratory viruses precipitate exacerbations of asthma and allergy-polarizing transcription factors are redox sensitive. PAR1 contributes to the pathogenicity of influenza A,^7^ PAR1 and Toll-like receptor 3 are both upregulated by respiratory virus infections,^8^ ATP promotes T_H_2 immunity, and P_2_X_7_ expression is upregulated in asthma.^5^ It will therefore be of interest to investigate the operational role of pannexons as a signaling nexus in allergic sensitization and the triggering of disease exacerbations.

The sensitivity of Toll-like receptor 3–mediated activation to argatroban or PAR1 antagonists (see Fig E2, *E*-*F*) suggests that events downstream of pannexon opening involve the endogenous activation of thrombin, creating a cyclical process. These findings reveal a surprising primary trigger for thrombin production that further emphasize its contribution to inflammatory lung responses. Although an oral thrombin inhibitor, albeit with bioavailability and protein binding which may preclude significant airway access from the systemic circulation, has only moderate improving effect on HDM-induced pathology in a murine model,^9^ our data suggest that it would be of interest to explore similar effects of ADIs, especially as these molecules have been optimized with the pharmaceutical credentials for inhaled delivery.

Additional information is available (see this article's Methods, Results, and References sections in the Online Repository at www.jacionline.org).

## Figures and Tables

**Fig 1:**
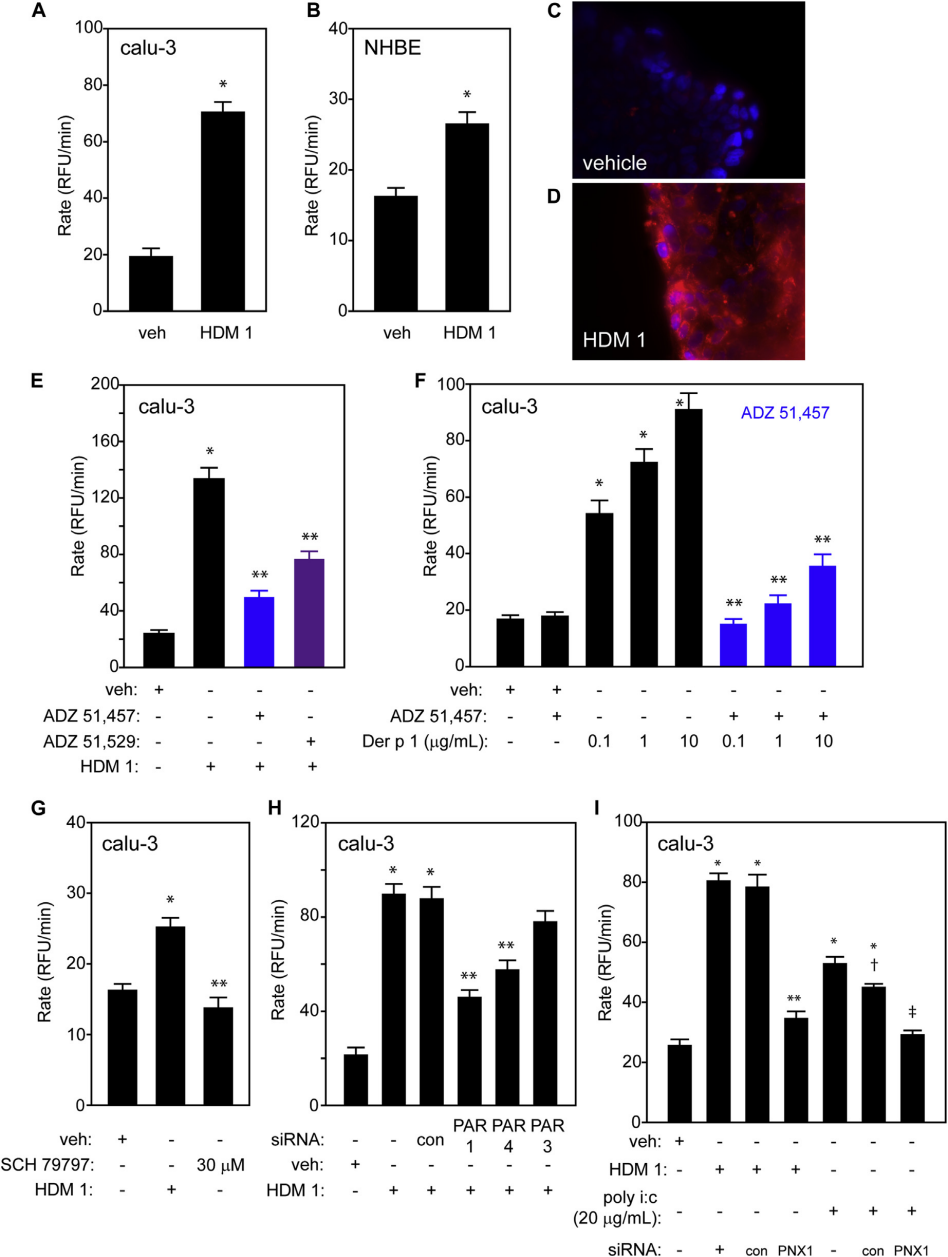
**A** and **B,** ROS production in calu-3 cells and primary cultures of human bronchial epithelial cells, respectively, following vehicle (veh) or HDM allergen treatment (*P < .001 vs veh control). **C** and **D,** MitoSOX red/NucBlue staining of calu-3 cells following veh or HDM1. **E,** Attenuation of HDM-induced ROS production by Der p 1 inhibitors (*P < .001 vs veh control; **P < .001 vs HDM). **F,** Inhibition of Der p 1 by ADZ 51,457 (*P < .001 vs veh control; **P < .001 vs corresponding Der p 1 concentration). **G** and **H,** Inhibition of HDM allergen-induced ROS production by the PAR1 antagonist SCH 79797 or by siRNA knockdown (*P < .001 vs veh; **P < .001 vs HDM 1 with or without control [con] transfection). **I,** ROS production by HDM allergens or by poly i:c is reduced in cells following knockdown of pannexin 1 (*P < .001 vs veh; **P < .001 vs HDM 1 with or without control transfection; †*P* < .05 vs poly i:c; ‡*P* < .001 vs poly i:c with or without control transfection *NHBE*, Normal human bronchial epithelial cells; *RFU*, relative fluorescence units.

**Fig 2:**
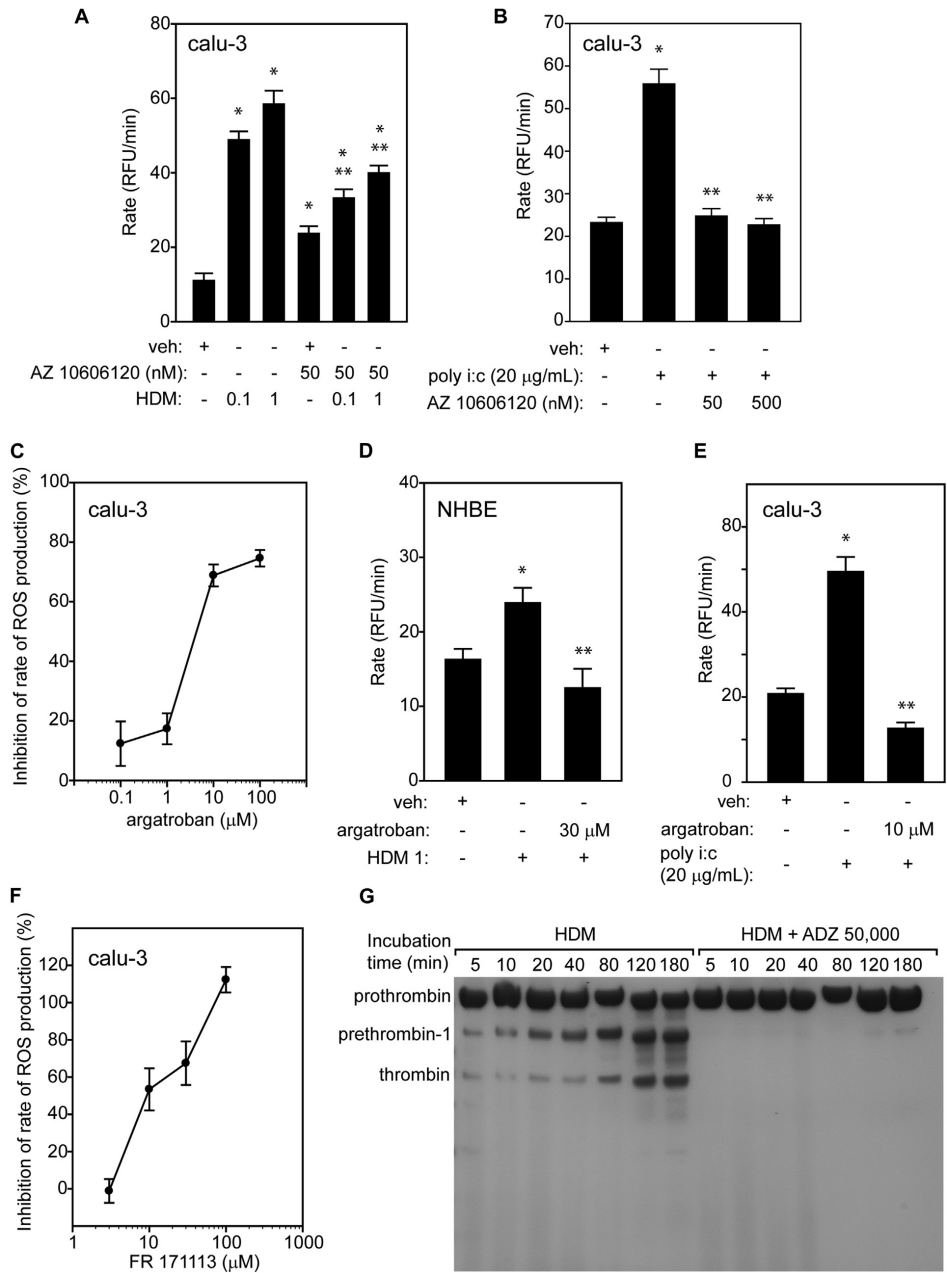
**A** and **B,** AZ 10606120 inhibits ROS production by HDM allergens or poly i:c (*P < .001 vs veh; **P < .001 vs HDM or poly i:c, respectively). **C** and **D,** Argatroban inhibits ROS generation by HDM allergens and by poly i:c (*P < .001 vs veh; **P < .001 vs HDM or poly i:c. **F,** Antagonism of poly i:c-dependent ROS production by PAR1 antagonist FR 171113 (*P* < .001 except at 3 μM). **G,** Time-dependent proteolysis of prothrombin 1 by mixed HDM allergens and its inhibition by ADZ 50,000. *NHBE*, Normal human bronchial epithelial cells; *RFU*, relative fluorescence units.

## Supplementary data

Online Repository text
