# Supplementary material for: Innate generation of thrombin and intracellular oxidants in airway epithelium by allergen Der p 1
Source: J Allergy Clin Immunol. 2016 Oct;138(4):1224–7. doi: 10.1016/j.jaci.2016.05.006 (PMC5052125; doi:10.1016/j.jaci.2016.05.006)
Supplement: Online Repository text [file mmc1.pdf]

SUPPLEMENTARY INFORMATION

Innate Generation of Thrombin and Intracellular Oxidants in Airway Epithelium by Allergen Der p 1

Jihui Zhang, PhD,[a,c] Jie Chen, MSc MB BS,[c] Kimberley Allen-Philbey, BSc, Chathuri Perera Baruhupolage, BSc, Theresa Tachie-Menson, BSc, Shannon C Mangat, BSc, David R Garrod, PhD,[b] & Clive Robinson, PhD

Institute for Infection & Immunity, St George's, University of London, London, UK. [a]Present address: State Key Laboratory of Microbial Resources, Institute of Microbiology, Chinese Academy of Sciences, Beijing, P.R. China. [b]Faculty of Life Sciences, University of Manchester, Manchester, UK.

[c]These authors contributed equally to this work.

Correspondence should be addressed to C.R. (c.robinson@sgul.ac.uk).

This work was supported by the Wellcome Trust (Award 087650, to CR).

Disclosure of potential conflict of interest: The authors declare that they have no conflicts of interest.

Abbreviations:

ADI:	Allergen Delivery Inhibitor
BzATP:	2′(3′)-O-(4-benzoylbenzoyl)adenosine 5′-triphosphate
DMSO:	Dimethyl sulfoxide
HBSS:	Hanks' balanced salt solution
HDM:	House dust mite
HEPES:	4-(2-hydroxyethyl)-1-piperazine ethanesulfonic acid
JAK:	Janus kinase
LC-MS-MS:	Liquid chromatography tandem mass spectrometry
MDA-5:	Melanoma differentiation associated protein-5
NHBE:	Normal human bronchial epithelial cells
PAR:	Protease-activated receptor
PBS:	Phosphate buffered saline
Poly i:c:	Polyinosinic:polycytidylic acid
RIG-I:	Retinoic acid inducible gene-I
ROS:	Reactive oxidant species
TLR:	Toll-like receptor

Materials and Methods

Chemicals and reagents

SCH 79797 (N^3-Cyclopropyl-7-[[4-(1-methylethyl)phenyl]methyl]-7*H*-pyrrolo[3,2-*f*]quinazoline-1,3-diamine dihydrochloride), FR 171113 (2-[3-(4-Chlorophenyl)-2-[(2,4-dichlorobenzoyl)imino]-4-oxo-5-thiazolidinylidene]-acetic acid methyl ester), tcY-NH2 (*trans*-cinnamoyl-YPGKF-NH_2), AC55541 ((2*E*)-2-[1-(3-Bromophenyl)ethylidene] α-(benzoylamino)-3,4-dihydro-4-oxo-1-phthalazineacetic acid hydrazide),TRAP-6 (SFLLRN), and AZ 10606120 (*N*-[2-[[2-[(2-Hydroxyethyl)amino]ethyl]amino]-5-quinolinyl]-2-tricyclo[3.3.1.13,7]dec-1-ylacetamide dihydrochloride) and (3*R*,4*R*)-4-Methyl-3-(methyl-7*H*-pyrrolo[2,3-*d*]pyrimidin-4-ylamino)-β-oxo-1-piperidinepropanenitrile citrate (CP 690550; Tofacitinib citrate) were all purchased from Tocris. ENMD-1068 (6-amino-1-[4-93-methyl-1-oxobutyl)-1-piperazinyl]-1-hexanone hydrochloride) was obtained from Enzo. Apixaban (4,5,6,7-tetrahydro-1-(4-methoxyphenyl)-7-oxo-6-[4-(2-oxo-1-piperidinyl)phenyl]-1H-pyrazolo[3,4-c]pyridine-3-carboxamide) was from Cayman Chemical. Argatroban monohydrate ((2R,4R)-1-[(2S)-5-[(aminoiminomethyl)amino]-1-oxo-2-[[(1,2,3,4-tetrahydro-3-methyl-8-quinolinyl)sulfonyl]amino]pentyl]-4-methyl-2-piperidinecarboxylic acid), BzATP (2′(3′)-O-(4-benzoylbenzoyl)adenosine 5′-triphosphate triethylammonium salt), AEBSF (4-(2-aminoethyl) benzenesulfonyl fluoride hydrochloride), acetovanillone, rotenone, cycloheximide, monensin, human thrombin and molecular weight markers were from Sigma-Aldrich. Human prothrombin (native, purified) was from Stratech Scientific Ltd. ADZ 50,000, ADZ 51,457 and ADZ 51,529, Allergen Delivery Inhibitors directed against the cysteine protease activity of Group 1 HDM allergens, were synthesized as described.[1] Polyinosinic:polycytidylic acid, 1.5-8 kb (InvivoGen), was used routinely, with comparative studies being made with a shorter form of poly i:c (0.2 – 1 kb) where stated.

Goat antibodies against human PAR1, PAR4 and pannexin-1 were from Santa Cruz Biotechnology. FITC or Alexa 488-conjugated secondary antibodies were from Sigma-Aldrich and Life Technologies. Fluorescent antibody labelling was performed conventionally. Dihydrorhodamine 123, MitoSOX red and NucBlue were from Life Technologies.

Cell culture media and reagents were from Life Technologies, Sigma-Aldrich, Lonza, or PAA. Transfection reagents and siRNA duplexes (typically mixtures of 3 target-specific 19-25 nt siRNAs or scrambled controls against no known targets) were from Santa Cruz Biotechnology.

Mixed, native HDM allergens in their natural proportions were prepared from laboratory cultures of *D. pteronyssinus* according to our standard procedures. Der p 1 content of the allergen extracts was determined by ELISA (Indoor Biotechnologies), while functional catalytic activity of Der p 1 was determined using a Der p 1-selective substrate. [2] HDM mixtures were normalized by reference to Der p 1 content expressed as $\mu g\ mL^{-1}$. Thus, HDM 1 refers to mixed HDM whose Der p 1 concentration is 1 $\mu g\ mL^{-1}$, and so forth. Native Der p 1, devoid of contaminant serine protease activity, was purified from these mixtures by ion-exchange, gel filtration and affinity chromatography according to in-house procedures. To ensure consistent proteolytic activity of Der p 1 in both natural mixtures and purified protein forms, experiments were conducted in the presence of 5mM L-cysteine. This tactic is justified given the presence of high concentrations of reducing agents in airway surface lining fluid.[3]

Cell culture and transfection

Calu-3, primary cultures of human bronchial epithelial cells, and NCI-H292 cells were cultured according to standard procedures, with the primary cultures being conducted in defined serum-free medium.[4-6] Experiments described in the accompanying letter were conducted in calu-3 cells or primary cultures of normal human bronchial epithelial cells (NHBE). Supplementary data in this document refer to experiments in NCI-H292 cells, calu-3 cells and NHBE. NHBE were cultured from grossly normal lung tissue removed during resection of bronchial carcinoma. Tissue recovery was performed under ethical approval and informed consent.

Intracellular ROS production was studied in cells plated into 96-well format on clear-bottomed black culture plates (Corning). Cells were washed and then loaded for 15 min at room temperature with dihydrorhodamine-123 (10 µM) in PBS, after which excess probe was removed by washing and the PBS replaced by HBSS containing 20 mM HEPES. Where appropriate, cells were then treated with inhibitors for 20 min at 37°C prior to the addition of HDM allergen or receptor agonists. Reactions were started by the addition of mixed HDM allergens or purified Der p 1 and maintained at 30°C under constant humidity in an Envision plate reader (Perkin Elmer) for the duration of the experiment. Fluorescence measurements were made every 5 min (excitation 485 nm, emission 535 nm) and the maximum rate of oxidant production determined from the progress curves (increase in fluorescence upon oxidation of dihydrorhodamine 123) recorded for each well over a 2.5 h period.

Fluorescent antibody labelling was conducted on cells grown on glass coverslips according to conventional procedure after methanol fixation. Live cells were stained with MitoSOX Red according to manufacturer instructions. Knockdown experiments with siRNA duplexes were performed according to the supplier protocol optimized for these experiments.

Prothrombin degradation

Mixed HDM allergens were dissolved in HBSS containing 20 mM HEPES and 5mM L-cysteine to produce a solution containing 5 µg mL^{-1} Der p 1. Eighteen microliters of this solution were mixed with 162 µL human prothrombin (final reaction concentration 20 µM) dissolved in 0.1M Tris.HCl, 150 mM NaCl, 5 mM $CaCl_2.2H_2O$ and containing 100 µM argatroban monohydrate to prevent autocatalysis. The mixture was incubated at 37°C and aliquots (14 µL) withdrawn into 1 µL ADZ 50,000 (20 mM in anhydrous DMSO) to inhibit further reaction. The samples were then analysed to identify the reaction products.

Statistical analyses

Analyses were performed using SigmaPlot v12. Data are shown as mean ± s.e.m (n=8) in single experiments which were replicated >3 times. Significance was determined using one-way analysis of variance with Newman-Keuls *post hoc* testing.

Results

Generation of intracellular ROS in airway epithelial cells and its inhibition

Exposure of airway epithelial cells to mixed HDM allergens resulted in the production of ROS, as was also observed on treatment with the P_2X_7 receptor agonist BzATP (**Fig E1**). The mitochondrial complex I inhibitor, rotenone, inhibited the response to HDM allergens, as did the NADPH oxidase (NOX) inhibitor acetovanillone (**Fig E1b,c**). Responses were suppressed by deferoxamine in a concentration-dependent manner leading to full inhibition, confirming that the recorded changes in fluorescence were due to ROS formation detected by dihydrorhodamine-123 (**Fig E1d**).

Generation of intracellular ROS in airway epithelial cells by HDM allergens is independent of allergens with serine protease activity and Der p 2

In order to establish whether the serine protease allergen repertoire of HDM contributed to ROS generation we examined the response to mixed HDM in which serine peptidase activity had been titrated using a molar excess of the irreversible serine protease inhibitor, AEBSF. Surplus AEBSF was removed from the allergen preparation by spin column treatment until no inhibitor could be detected in the eluate by LC-MS-MS analysis (Shimadzu Prominence/ABI Sciex 3200 Q trap) and the efficacy of titration confirmed by serine protease activity assay. **Fig E2a** shows that responses of calu-3 cells to AEBSF-titrated HDM allergens did not differ significantly from the non-titrated control HDM allergen preparation, indicating that serine peptidase allergens (HDM Group 3, 6 and 9, all of which are tryptic or chymotryptic serine peptidases and targets of AEBSF[7]) make little contribution to ROS generation.

In the next series of experiments we investigated the effect of purified Der p 2 as an archetype of Group 2 HDM allergens. These Group 2 allergens are clinically significant and are homologues of the MD-2 component of TLR4 which is implicated in orchestrating other innate responses to HDM allergens[7, 8]. As shown in **Fig E2b**, whereas purified Der p 1 was a potent stimulant of ROS production Der p 2 was without effect. Collectively, these data further support the view that among natural HDM allergens the primary initiators of intracellular ROS generation are the Group I cysteine peptidases (see the letter associated with this online repository information).

Intracellular ROS generation is primarily dependent on PAR1 and PAR4

Given literature precedent suggesting a major role for PAR2 in cytokine release due to the proteolytic activity of HDM allergens[9-11], we electively examined whether ROS generation depended upon PAR cleavage. Surprisingly, the PAR2 antagonist ENMD-1068 produced a weak, partial inhibition of the response to mixed HDM allergens, whereas the PAR1 antagonists SCH 79797 and FR 171113, which are structurally unrelated to each other, and the PAR4 antagonist tcY-NH_2 more effectively abrogated ROS production (**Fig E3a-c**). PAR1 and PAR4 are both expressed in calu-3 cells, as confirmed by fluorescent antibody labelling (**Fig E3d,e**), and the effective blockade of responses by FR 171113 confirmed in a second cell line (**Fig E3f**).

Desensitization experiments were undertaken as a further means to investigate the receptors mediating ROS production. For these studies, cells were exposed to PAR agonists (TRAP-6 for PAR1, AC 55541 for PAR2, AY-NH_2 for PAR4) for 20 min at 37°C before stimulating the cells with mixed HDM allergens. Forerunner studies indicated that only poor desensitization of PAR4 was achievable with AY-NH_2 which precluded a full appreciation of its role using this approach (data not shown), but despite this the desensitization strategy broadly confirmed the significant role of PAR1 and the lesser

role of PAR2 (**Fig E3g**). In both calu-3 and NCI-H292 cells siRNA knockdown of PAR2 achieved only moderate attenuation of responses to HDM allergens (**Fig E3h,i**). Nothwithstanding the caveat concerning the extent of PAR4 desensitization, these data (**Fig E3a-c, g-i**) indicate that the combined significance of PAR1 and PAR4 as the transducers of ROS production outweighs the role of PAR2 and provide robust independent verification of knockdown studies using siRNA (see the letter associated with this online repository information).

Poly i:c stimulates intracellular ROS production through activation TLR3, MDA5 and RIG-I and is dependent upon protein synthesis and export

As disclosed in the letter associated with this online repository information, use of poly i:c as a surrogate for viral RNA resulted in ROS production by airway epithelial cells. Activation of ROS production by high molecular weight poly i:c was dependent on TLR3 and MDA5 (**Fig E4a,b**). However, when poly i:c with a lower molecular weight was used the responses were blunted by knockdown of either MDA5 or RIG-I, consistent with the nucleotide length-dependencies of these pattern recognition receptors (**Fig E4c**).

In contrast to activation by the HDM allergen mixture, ROS generation by poly i:c was inhibited by pre-treatment of cells with cycloheximide, monensin or CP 690550, indicating an obligatory role for protein synthesis, protein transport and the activation of JAK-based signaling (**Fig 4d-g**). These dissociations are indicative of events upstream from the convergence point in the signaling pathways activated by poly i:c and Der p 1 which we speculate is proximal to the operation of pannexons.

Intracellular ROS generation by HDM allergen mixture involves the activation of thrombin but not Factor X

As was the case in calu-3 cells, argatroban was a concentration-dependent inhibitor of ROS production in NCI-H292 cells stimulated by mixed HDM allergens (**Fig E5a**). To provide further confirmation that thrombin formation by Der p 1 was responsible for activating ROS production, we next demonstrated the inhibition of calu-3 cell responses to Der p 1 by argatroban **Fig E5b**. Consistent with ROS production by Der p 1 and poly i:c being dependent upon PAR1 and PAR4, ROS generation was elicited by exogenous thrombin, although desensitization occurred readily, resulting in a reduction of response with increasing thrombin concentration (**Fig E5c**). Significantly, the Factor Xa inhibitor apixaban did not affect the response to HDM allergens, thus unambiguously indicating the formation of thrombin without activation of the full coagulation cascade (**Fig E5d**).

Inhibition of intracellular ROS generation by knockdown of pannexons or allosteric modulation by AZ 10606120

AZ 10606120, a negative allosteric modulator of P_2X_7 receptors, inhibited ROS generation by mixed HDM allergens in NCI-H292 cells consistent with observations in calu-3 cells (**Fig E6a**). These data imply the extracellular release of ATP following stimulation by Der p 1. This modulator prevented ROS generation by exogenous thrombin, consistent with ATP release being downstream of PAR activation (**Fig E6b**). Confirming the critical role of pannexons in ROS production revealed by other experiments described in the letter associated with this online repository material, siRNA knockdown of pannexin-1 strongly attenuated the response to HDM allergens in NCI-H292 cells (**Fig E6c**).

Supplementary Figures

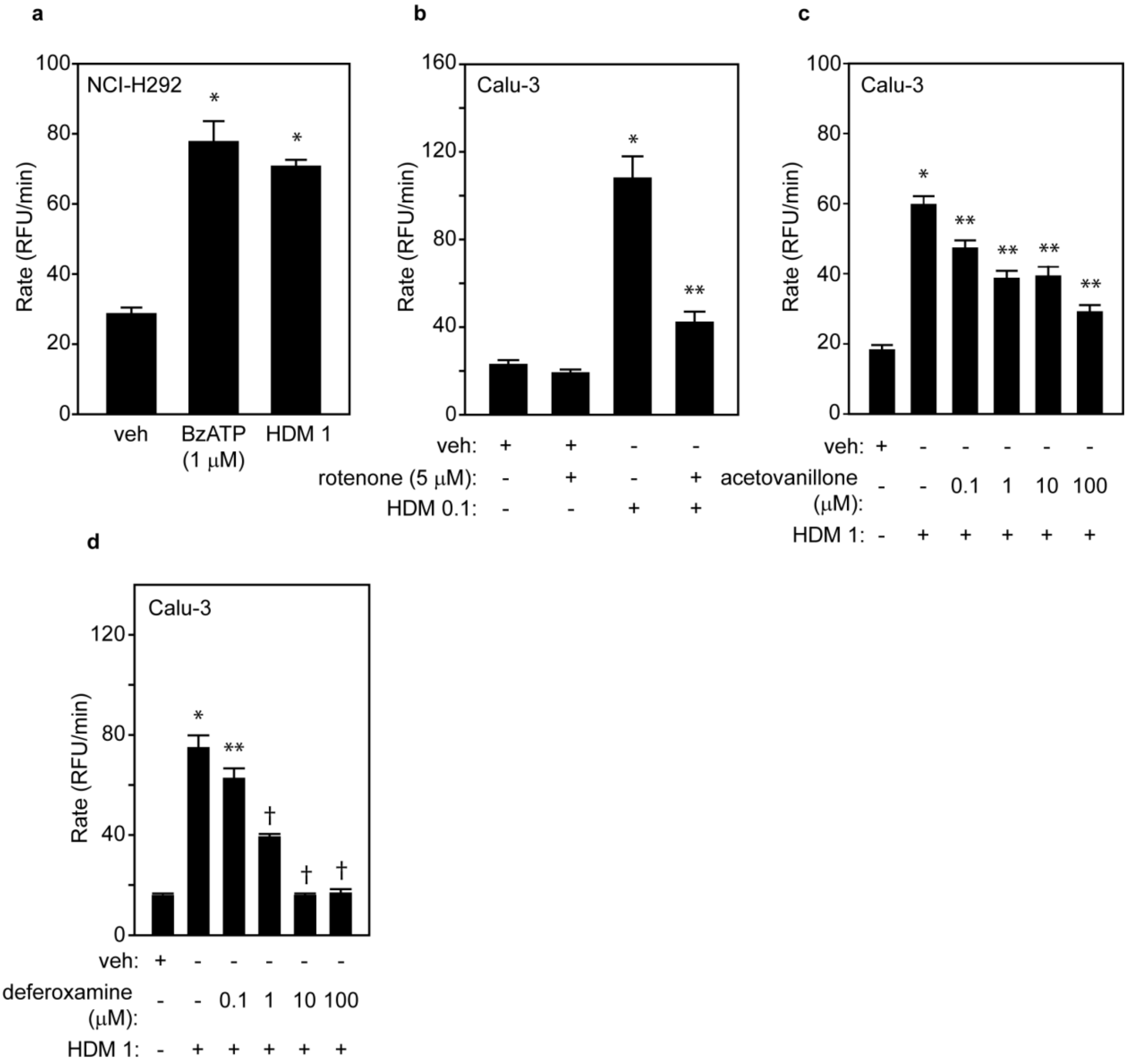

Figure E1 Generation of intracellular ROS in airway epithelial cells and its inhibition. (**a**) Effects of vehicle (veh), BzATP or mixed house dust mite allergens containing Der p 1 at 1 μg mL^{-1} (HDM 1) on ROS generation in NCI-H292 cells. *P<0.001 *v* veh. (**b**) Effect of rotenone on ROS generation in calu-3 cells treated with veh or mixed house dust mite allergens containing Der p 1 at 0.1 μg mL^{-1} (HDM 0.1). *P<0.001 *v* veh (with or without rotenone), **P<0.001 *v* HDM 0.1. (**c**) Effect of acetovanillone on ROS generation in calu-3 cells treated with veh or HDM 1. *P<0.001 *v* veh, **P<0.001 v HDM 1. (**d**) Effect of deferoxamine on ROS generation in calu-3 cells treated with veh or HDM 1. *P<0.001 *v* veh, **P<0.05 *v* HDM 1, †P<0.001 *v* HDM 1.

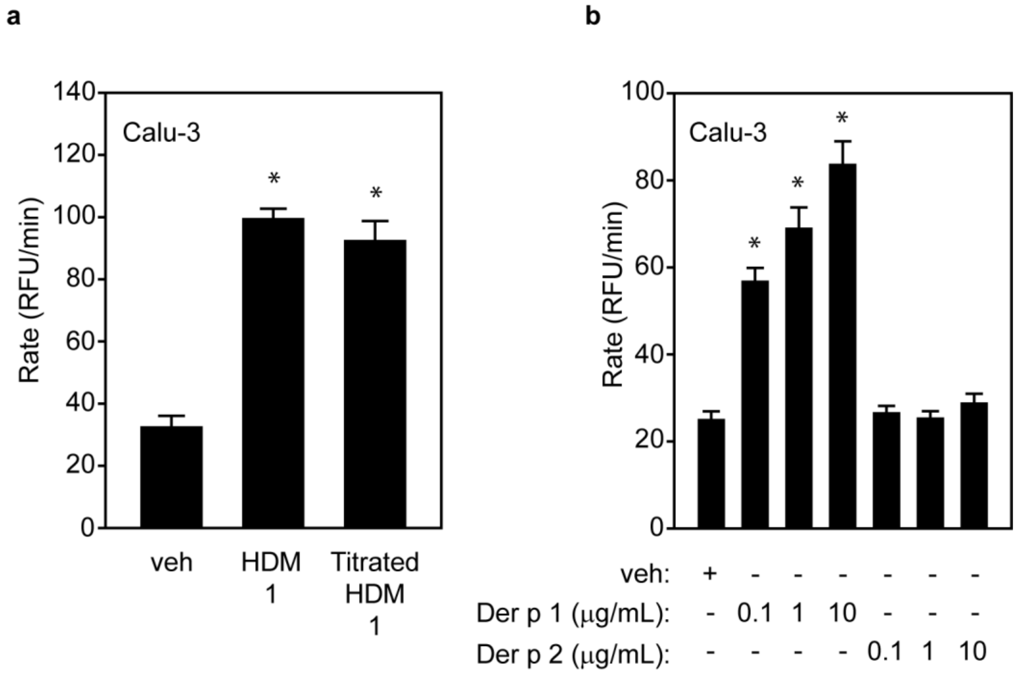

Figure E2 Generation of intracellular ROS in airway epithelial cells by HDM allergens is independent of allergens with serine protease activity and Der p 2. (**a**) Comparison of ROS production in calu-3 cells by vehicle (veh), HDM 1 and HDM 1 titrated with the serine peptidase inhibitor AEBSF. *P<0.001 *v* veh. No significant difference exists between HDM 1 and HDM 1 titrated with AEBSF. (**b**) Comparison of ROS production in calu-3 cells by purified Der p 1 and Der p 2. *P<0.001 *v* veh. No significant difference exists between Der p 2 (0.1 – 10 μg mL^{-1}) and veh.

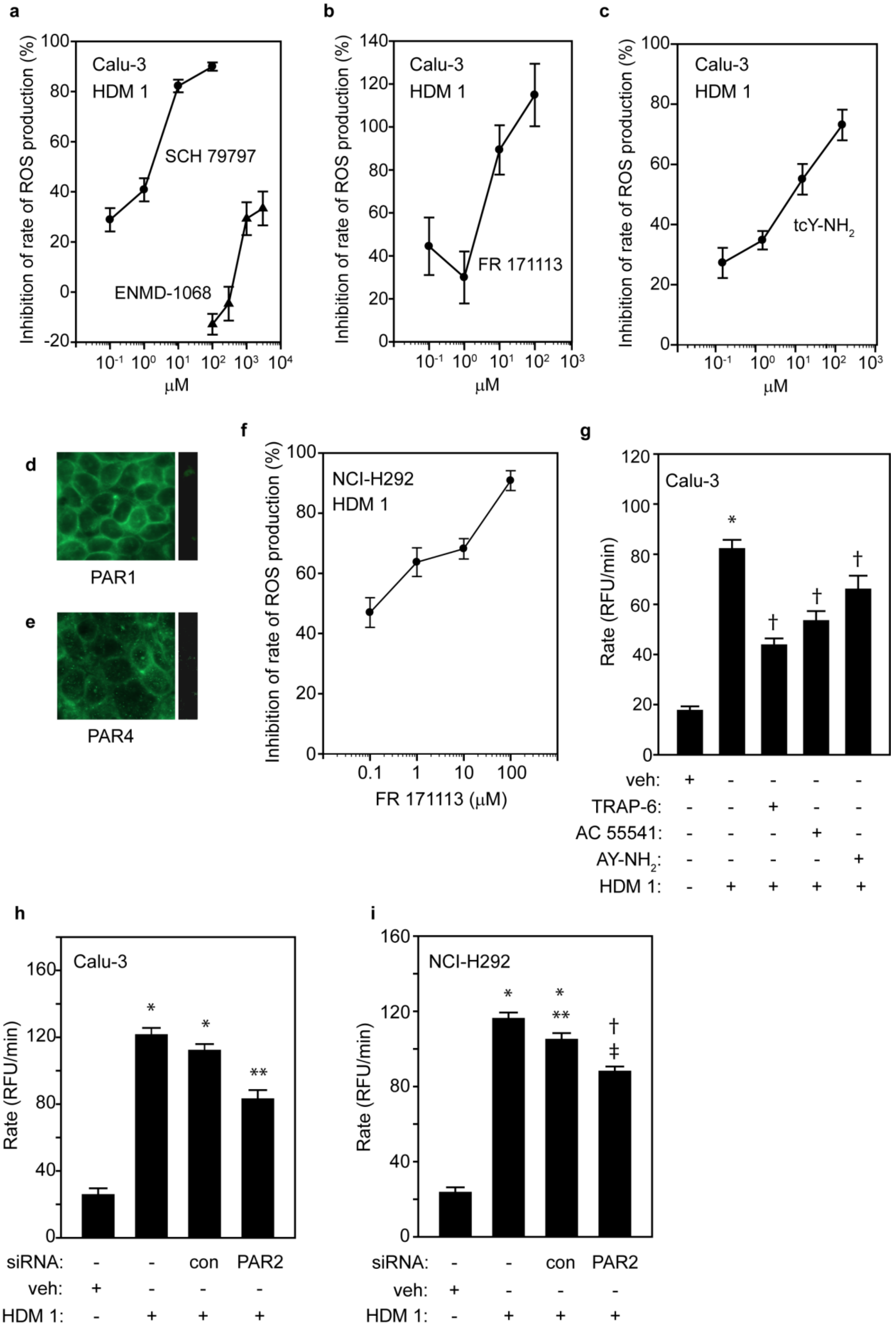
a
Calu-3
HDM 1
SCH 79797
ENMD-1068
Inhibition of rate of ROS production (%)
μM
b
Calu-3
HDM 1
FR 171113
Inhibition of rate of ROS production (%)
μM
c
Calu-3
HDM 1
tcY-NH2
Inhibition of rate of ROS production (%)
μM
d
PAR1
e
PAR4
f
NCI-H292
HDM 1
Inhibition of rate of ROS production (%)
FR 171113 (μM)
g
Calu-3
Rate (RFU/min)
veh:
TRAP-6:
AC 55541:
AY-NH2:
HDM 1:
h
Calu-3
Rate (RFU/min)
siRNA: - - con PAR2
veh: + - - -
HDM 1: - + + +
i
NCI-H292
Rate (RFU/min)
siRNA: - - con PAR2
veh: + - - -
HDM 1: - + + +

Figure E3 (previous page) Intracellular ROS production in human airway epithelial cells by HDM allergens is primarily dependent on PAR1 and PAR4. (**a-c**) Effects of PAR1, PAR2 and PAR4 antagonism on ROS production by calu-3 cells stimulated by mixed HDM allergens. (**d,e**) Immunofluorescence labelling of PAR1 and PAR4 (strip images show negative controls). (**f**) Effect of PAR1 antagonist FR 171113 on ROS generation by NCI-H292 cells stimulated by HDM allergen mixture. Inhibition was significant (P<0.001) at all concentrations tested. (**g**) Effects of PAR receptor agonist pre-treatment (PAR 1: TRAP-6 (100 µM); PAR2:AC 55541 (10 µM), and PAR4:AY-NH_2 (100 µM)) on the generation of ROS in calu-3 cells stimulated with mixed HDM allergens. Agonists were incubated with cells for 20 min prior to addition of HDM 1. *P<0.001 *v* veh pre-treatment and sham stimulation, [†]P<0.001 *v* HDM 1. (**h**) Effect of PAR2 siRNA on response to HDM 1 in calu-3 cells. *P<0.001 *v* vehicle; **P<0.001 *v* HDM with and without control (con) transfection. (**i**) Effect of siRNA knockdown of PAR2 on the ability of HDM 1 mixture to elicit intracellular ROS production in NCI-H292 cells. *P<0.001 *v* veh, **P<0.05 *v* HDM 1, [†]P<0.001 *v* HDM 1, [‡]P<0.001 *v* siRNA control transfection.

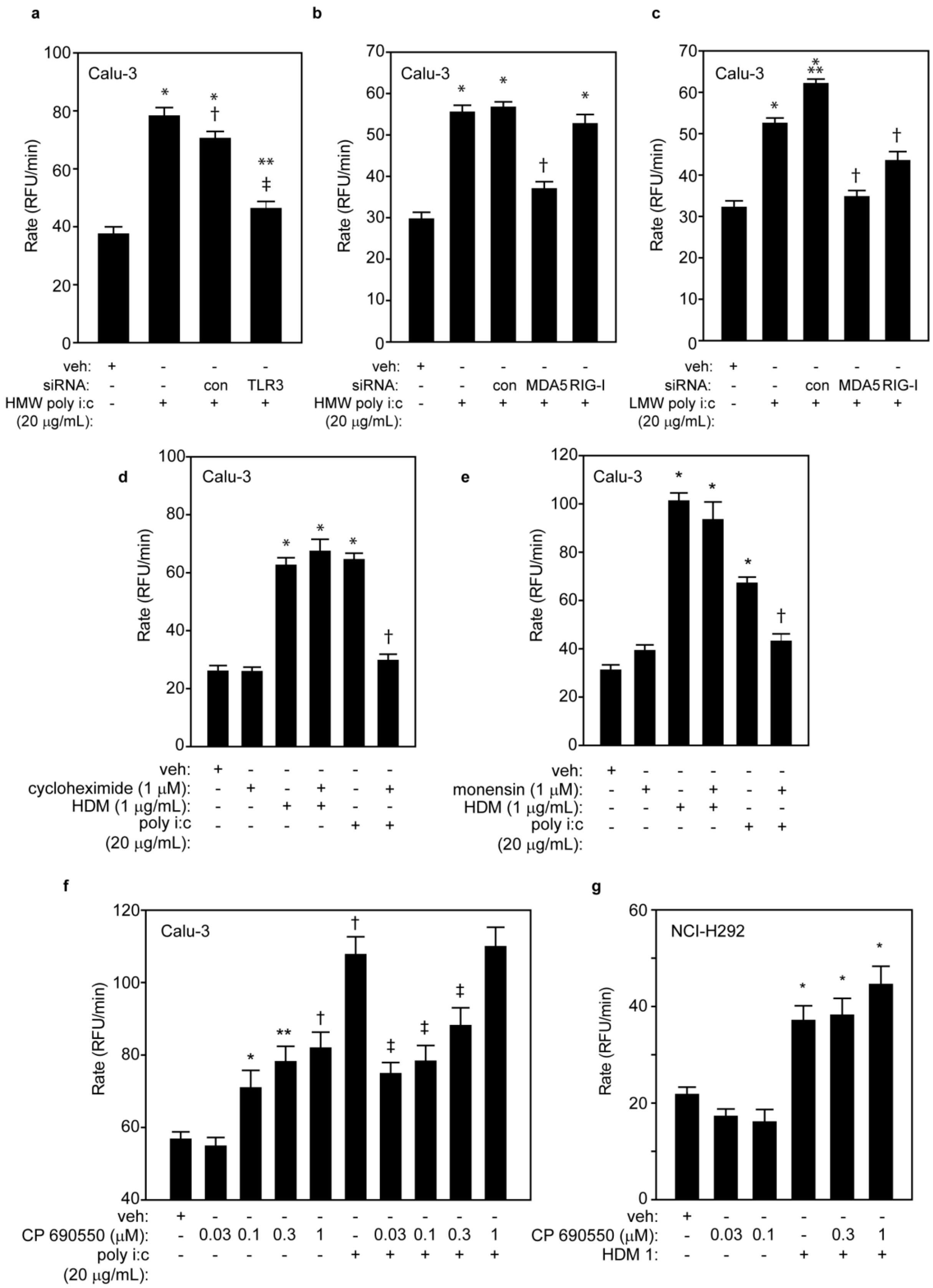
a
Calu-3
Rate (RFU/min)
0
20
40
60
80
100
*
*
†
**
‡
veh: + - - -
siRNA: - - con TLR3
HMW poly i:c (20 μg/mL): - + + +
b
Calu-3
Rate (RFU/min)
0
10
20
30
40
50
60
70
*
*
†
*
veh: + - - - -
siRNA: - - con MDA5 RIG-I
HMW poly i:c (20 μg/mL): - + + + +
c
Calu-3
Rate (RFU/min)
0
10
20
30
40
50
60
70
*
*
**
†
†
veh: + - - - -
siRNA: - - con MDA5 RIG-I
LMW poly i:c (20 μg/mL): - + + + +
d
Calu-3
Rate (RFU/min)
0
20
40
60
80
100
*
*
*
†
veh: + - - - - -
cycloheximide (1 μM): - + - + - +
HDM (1 μg/mL): - - + + - -
poly i:c (20 μg/mL): - - - - + +
e
Calu-3
Rate (RFU/min)
0
20
40
60
80
100
120
*
*
*
†
veh: + - - - - -
monensin (1 μM): - + - + - +
HDM (1 μg/mL): - - + + - -
poly i:c (20 μg/mL): - - - - + +
f
Calu-3
Rate (RFU/min)
40
60
80
100
120
*
**
†
†
‡
‡
‡
veh: + - - - - - - - - -
CP 690550 (μM): - 0.03 0.1 0.3 1 - 0.03 0.1 0.3 1
poly i:c (20 μg/mL): - - - - - + + + + +
g
NCI-H292
Rate (RFU/min)
0
20
40
60
*
*
*
veh: + - - - - -
CP 690550 (μM): - 0.03 0.1 - 0.3 1
HDM 1: - - - + + +

Figure E4 (Previous page) Poly i:c stimulates intracellular ROS production through activation TLR3, MDA5 and RIG-I and is dependent upon protein synthesis and export. (**a**) siRNA knockdown of TLR3 attenuates the response to high molecular weight poly i:c. *P<0.001 *v* veh, †P<0.05 *v* poly i:c, **P<0.01 *v* veh, ‡P<0.001 *v* transfection control. (**b**) Knockdown of MDA5, but not RIG-I, attenuates the response to high molecular weight poly i:c, whereas (**c**) knockdown of either inhibits the response to low molecular weight poly i:c. *P<0.001 *v* veh control, **P<0.001 *v* stimulated non-transfected cells; †P<0.001 *v* stimulated cells with control (con) transfection. (**d**) Effect of cycloheximide on ROS generation by mixed HDM allergens or poly i:c. *P<0.001 *v* veh control and unstimulated cells pre-treated with cycloheximide, †P<0.001 *v* poly i:c. (**e**) As **d**, but using monensin. (**f**) Effect of CP 690550 on the production of ROS in the absence or presence of poly i:c. *P<0.05 *v* veh, **P<0.01 *v* veh, †P<0.001 *v* veh, ‡P<0.001 *v* poly i:c. (**g**) Lack of inhibition of ROS production in cells stimulated with mixed HDM allergens. *P<0.001 *v* veh.

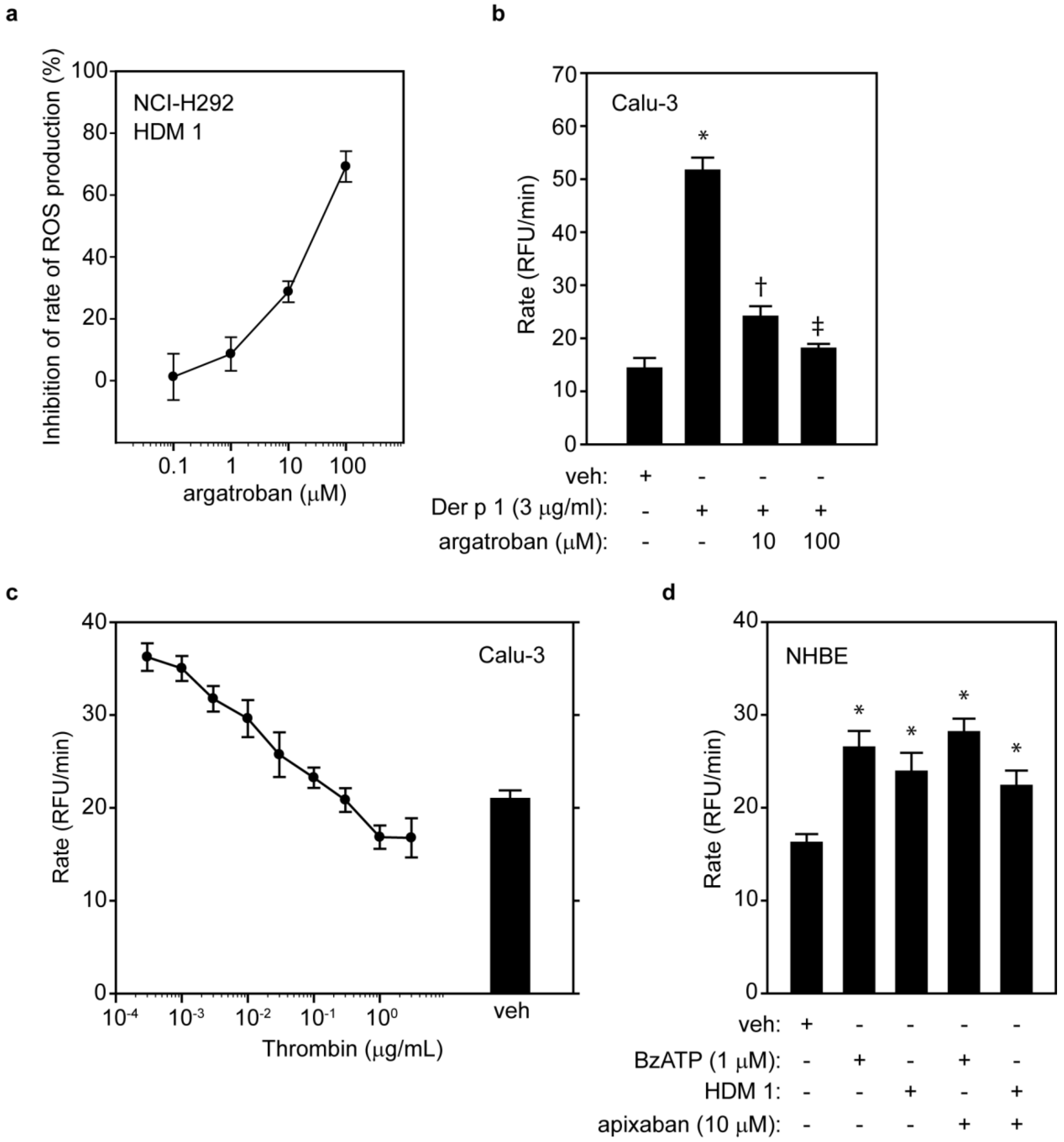

Figure E5 Intracellular ROS generation by HDM allergen mixture involves the activation of thrombin but not Factor X. (**a**) Effect of argatroban on ROS production in NCI-H292 cells stimulated with mixed HDM allergens. Inhibition was statistically significant (P<0.001) at 10 and 100 μM. (**b**) Effect of argatroban on ROS production in calu-3 cells treated with vehicle (veh) or purified Der p 1. *P<0.001 *v* veh, †P<0.001 v Der p 1, ‡P<0.05 *v* 10 μM argatroban. (**c**) Effect of veh or exogenous thrombin on ROS production in calu-3 cells. The effects of thrombin were statistically significant (P<0.001) compared to veh control over the range 0.3 – 10 ng mL^{-1}. (**d**) Effect of the factor Xa inhibitor, apixaban, on intracellular ROS generation in primary cultures of human airway epithelial cells

stimulated with veh, BzATP or HDM allergen mixture. *P<0.001 *v* veh. Apixaban did not significantly affect the responses to either HDM 1 or BzATP.

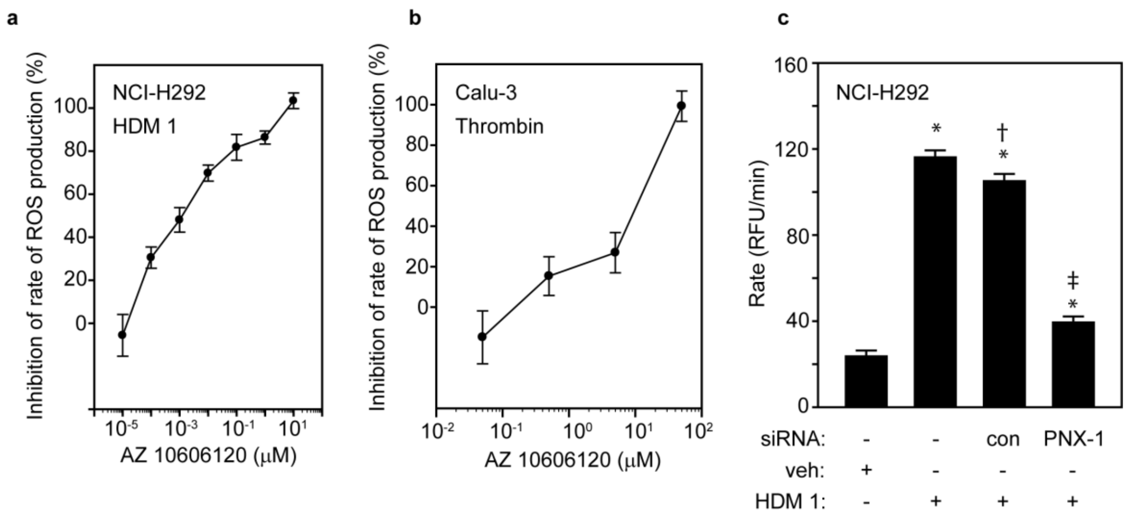

Figure E6 Inhibition of intracellular ROS generation by knockdown of pannexons or allosteric modulation by AZ 10606120. (**a**) Effect of AZ 10606120 on ROS production stimulated by HDM allergen mixture in NCI-H292 cells Inhibition was statistically significant (P<0.001) at all concentrations tested. (**b**) Effect of AZ 10606120 on ROS production stimulated by thrombin (3 ng mL^{-1}) in calu-3 cells. Thrombin increased ROS production from 24.0 ± 1.5 to 47.1 ± 1.6 RFU min^{-1} in the absence of AZ 10606120 (P<0.001). The inhibition by AZ 10606120 was significant (P<0.001) at 50 µM. (**c**) Effect of knockdown of pannexin-1 by siRNA duplexes on the ability of HDM allergen mixture to elicit intracellular ROS production in NCI H292 cells. The effects of control (con) scrambled duplexes are shown for comparison with the target siRNA treatment. *P<0.001 *v* vehicle, †P<0.001 *v* HDM 1, ‡P<0.001 *v* HDM 1 with control (con) siRNA transfection.

References

1. Newton GK, Perrior TR, Jenkins K, Major MR, Key RE, Stewart MR, et al. The discovery of potent, selective, and reversible inhibitors of the house dust mite peptidase allergen Der p 1: an innovative approach to the treatment of allergic asthma. J Med Chem 2014; 57:9447-62.

2. Zhang J, Hamilton JM, Garrod DR, Robinson C. Interactions between mature Der p 1 and its free prodomain indicate membership of a new family of C1 peptidases. Allergy 2007; 62:1302-9.

3. Comhair SAA, Erzurum SC. Redox control of asthma: Molecular mechanisms and therapeutic opportunities. Antioxidants & Redox Signaling 2010; 12:93-124.

4. Winton HL, Wan H, Cannell MB, Gruenert DC, Thompson PJ, Garrod DR, et al. Cell lines of pulmonary and non-pulmonary origin as tools to study the effects of house dust mite proteinases on the regulation of epithelial permeability. Clin Exp Allergy 1998; 28:1273-85.

5. Wan H, Winton HL, Soeller C, Stewart GA, Thompson PJ, Gruenert DC, et al. Tight junction properties of the immortalized human bronchial epithelial cell lines Calu-3 and 16HBE14o^{-}. Eur Respir J 2000; 15:1058-68.

6. Wan H, Winton HL, Soeller C, Tovey ER, Gruenert DC, Thompson PJ, et al. Der p 1 facilitates transepithelial allergen delivery by disruption of tight junctions. J Clin Invest 1999; 104:123-33.

7. Stewart GA, Richardson JP, Zhang J, Robinson C. The structure and function of allergens. In: Adkinson NF, Bochner BS, Burks AW, Busse WW, Holgate ST, Lemanske RF, et al., editors. Middleton's Allergy - Principles and Practice. Philadelphia Elsevier Saunders; 2014. p. 398-429.

8. Trompette A, Divanovic S, Visintin A, Blanchard C, Hegde RS, Madan R, et al. Allergenicity resulting from functional mimicry of a Toll-like receptor complex protein. Nature 2009; 457:585-8.

9. Asokananthan N, Graham PT, Stewart DJ, Bakker AJ, Eidne KA, Thompson PJ, et al. House dust mite allergens induce proinflammatory cytokines from respiratory epithelial cells: The cysteine protease allergen, Der p 1, activates protease-activated receptor (PAR)-2 and inactivates PAR-1. J Immunol 2002; 169:4572-8.

10. King C, Brennan S, Thompson PJ, Stewart GA. Dust mite proteolytic allergens induce cytokine release from cultured airway epithelium. J Immunol 1998; 161:3645-51.

11. Robinson C, Zhang J, Newton GK, Perrior TR. Nonhuman targets in allergic lung conditions. Future Med Chem. 2013; 5:147-61.
